# Saliva for Precision Dosing of Antifungal Drugs: Saliva Population PK Model for Voriconazole Based on a Systematic Review.

**DOI:** 10.3389/fphar.2020.00894

**Published:** 2020-06-12

**Authors:** Hannah Yejin Kim, Anne-Grete Märtson, Erwin Dreesen, Isabel Spriet, Sebastian G. Wicha, Andrew J. McLachlan, Jan-Willem Alffenaar

**Affiliations:** ^1^Sydney Pharmacy School, Faculty of Medicine and Health, The University of Sydney, Camperdown, NSW, Australia; ^2^Department of Pharmacy, Westmead Hospital, Westmead, NSW, Australia; ^3^Marie Bashir Institute of Infectious Diseases and Biosecurity, The University of Sydney, Camperdown, NSW, Australia; ^4^University of Groningen, University Medical Center Groningen, Department of Clinical Pharmacy and Pharmacology, Groningen, Netherlands; ^5^Department of Pharmaceutical and Pharmacological Sciences, Clinical Pharmacology and Pharmacotherapy, KU Leuven, Leuven, Belgium; ^6^Pharmacy Department, University Hospitals Leuven, Leuven, Belgium; ^7^Department of Clinical Pharmacy, Institute of Pharmacy, University of Hamburg, Hamburg, Germany

**Keywords:** saliva, oral fluid, therapeutic drug monitoring, precision dosing, antifungal drug, voriconazole, population pharmacokinetic model

## Abstract

Precision dosing for many antifungal drugs is now recommended. Saliva sampling is considered as a non-invasive alternative to plasma sampling for therapeutic drug monitoring (TDM). However, there are currently no clinically validated saliva models available. The aim of this study is firstly, to conduct a systematic review to evaluate the evidence supporting saliva-based TDM for azoles, echinocandins, amphotericin B, and flucytosine. The second aim is to develop a saliva population pharmacokinetic (PK) model for eligible drugs, based on the evidence. Databases were searched up to July 2019 on PubMed^®^ and Embase^®^, and 14 studies were included in the systematic review for fluconazole, voriconazole, itraconazole, and ketoconazole. No studies were identified for isavuconazole, posaconazole, flucytosine, amphotericin B, caspofungin, micafungin, or anidulafungin. Fluconazole and voriconazole demonstrated a good saliva penetration with an average S/P ratio of 1.21 (± 0.31) for fluconazole and 0.56 (± 0.18) for voriconazole, both with strong correlation (r = 0.89–0.98). Based on the evidence for TDM and available data, population PK analysis was performed on voriconazole using Nonlinear Mixed Effects Modeling (NONMEM 7.4). 137 voriconazole plasma and saliva concentrations from 11 patients (10 adults, 1 child) were obtained from the authors of the included study. Voriconazole pharmacokinetics was best described by one-compartment PK model with first-order absorption, parameterized by clearance of 4.56 L/h (36.9% CV), volume of distribution of 60.7 L, absorption rate constant of 0.858 (fixed), and bioavailability of 0.849. Kinetics of the voriconazole distribution from plasma to saliva was identical to the plasma kinetics, but the extent of distribution was lower, modeled by a scale factor of 0.5 (4% CV). A proportional error model best accounted for the residual variability. The visual and simulation-based model diagnostics confirmed a good predictive performance of the saliva model. The developed saliva model provides a promising framework to facilitate saliva-based precision dosing of voriconazole.

## Introduction

Invasive fungal infections can compromise clinical outcomes in critically-ill or immunocompromized patients receiving chemotherapy, solid organ or bone marrow transplant, or with diabetes and chronic obstructive pulmonary disease (COPD) (Ashbee et al., 2014). Significant pharmacokinetic variability resulting in suboptimal or toxic effects from antifungal drugs may effect treatment outcome. Prompt and successful individualized treatment is imperative, and optimal exposure and attainment of relevant pharmacokinetic-pharmacodynamic (PK/PD) target is warranted, often ensured by precision dosing.

With increasing evidence for dose-exposure-response relationships, US and European guidelines recommend therapeutic drug monitoring (TDM) for selected antifungal drugs including voriconazole, posaconazole, itraconazole (Ashbee et al., 2014; Patterson et al., 2016; Maertens et al., 2018; Warris et al., 2019), and flucytosine (Ashbee et al., 2014). Voriconazole steady-state trough concentrations <1–2 mg/L and >4–5.5 mg/L are associated with treatment failures and toxicity, respectively, further complicated by non-linear pharmacokinetics for this drug (Troke et al., 2011; Hamada et al., 2012; Pascual et al., 2012; Dolton et al., 2012b). Genetic polymorphisms in *CYP2C19* can result in up to three-fold increase in voriconazole exposure (Scholz et al., 2009; Lee et al., 2012). Itraconazole and posaconazole suspension demonstrate formulation and pH-dependent absorption and highly variable PK, posing a risk for suboptimal exposure (Barone et al., 1993; Van de Velde et al., 1996; van der Elst et al., 2015; Oh et al., 2020), which is associated with breakthrough infections (Glasmacher et al., 1999; Dolton et al., 2012a) and lower response rates (Walsh et al., 2007; van der Elst et al., 2015). TDM is recommended, with a target itraconazole trough concentration of 0.5–1 mg/L, and posaconazole trough of >0.7 mg/L (prophylaxis) (Jang et al., 2010; Tonini et al., 2012) or >1–1.5mg/L (treatment) (Ashbee et al., 2014). Given the wide range of MICs for some fungal pathogens such as *Aspergillus fumigatus*, and resistance-prone genetic mutations (Mavridou et al., 2010), interpretation of exposure-response relationship in the context of phenotypic and genotypic factors are also important in precision dosing. Flucytosine TDM is recommended based on the exposure-toxicity (myelotoxicity and hepatotoxicity) relationship (Stamm et al., 1987; Ashbee et al., 2014). The suggested trough target is 20–40 mg/L, and peak concentration <100 mg/L to minimize the toxicity (Ashbee et al., 2014). There is a lack of clinical need for routine TDM of fluconazole, echinocandins and (liposomal) amphotericin B, and isavuconazole (Ashbee et al., 2014; Patterson et al., 2016; Andes et al., 2018; Maertens et al., 2018; Warris et al., 2019).

Alongside the considerations for TDM, alternative sampling strategies are important to facilitate precision dosing of the antifungal drugs, especially to avoid invasive blood sampling in fragile, paediatric, or ambulatory patients, to reduce turnaround times and to enable availability of point-of-care devices using the alternative matrix.

“Salivary concentrations of antifungals may be useful for therapeutic drug monitoring”, was a statement made by Force and Nahata (1995). This, in addition to established dose-exposure-response relationships, provides a rationale for saliva-based TDM for these drugs (Glasmacher et al., 1999; Walsh et al., 2007; Dolton et al., 2012a; Dolton et al., 2012b).

Yet, to date, there are no population PK models describing the PK of antifungal drugs in saliva, and thus, guidance on saliva-based dosing is lacking.

This systematic review aims to summarize the available evidence on saliva pharmacokinetics of antifungals, including salivary distribution and correlation between saliva-plasma (S/P) concentrations. The second aim is to develop a saliva population PK model for the selected antifungal drug from published data to provide a framework for saliva-based precision dosing.

## Methods

### Systematic Literature Review

PubMed^®^ and Embase^®^ were searched using the following terms; “fluconazole or voriconazole or itraconazole or ketoconazole or isavuconazole or posaconazole or flucytosine or amphotericin B or caspofungin or micafungin or anidulafungin” (all fields) AND “saliva” (all fields) or “oral fluid” (keyword) for a period covering Jan 1947–July 2019. The references from each database were imported into a reference manager (Endnote^®^), and duplicates were removed. Titles and abstracts were screened, and articles with non-relevant topics were excluded. Eligible full text articles were screened and excluded if they contain non-human data, reviews, non-English, or no salivary PK data. Studies were included if conducted in humans and presented saliva/plasma time-concentration data. A reference list of review articles was also screened for potentially relevant studies. Saliva and plasma PK data including C_max_, AUC, S/P ratio, correlation coefficient, as well as dose regimens, sampling method, saliva assays and study group characteristics, were extracted from the included studies. The whole process of database search, in/exclusion process, and data extraction was repeated independently by a second person. Discrepancies were resolved by discussion.

### Population PK Analysis

Antifungal drugs for the population PK analyses were selected based on the supporting evidence for saliva TDM and data availability of paired S/P concentrations from published studies. The authors of the selected studies were contacted for individual patient data to be included in the model development. Ethics approvals for relevant studies were previously obtained by the authors.

Population PK analysis was applied to the obtained S/P drug concentration-time data using NONMEM 7.4 (Icon Development Solutions, Ellicott City, MD, USA), executed *via* Perl-speaks NONMEM (PsN 4.9.0) (Lindbom et al., 2004; Lindbom et al., 2005) with auxiliary graphical and visual interfaces, Pirana 2.9.9 (Keizer et al., 2011), and Xpose 4.5.3/R (Jonsson and Karlsson, 1999). The ADVAN 13 subroutine was used for parameterization of the pharmacokinetic compartments using differential equations. Firstly, a stable plasma structural PK model was built using only plasma drug concentration-time data. This was done by fitting one- and two-compartment models to the plasma concentration data, testing different error models (additive, proportional, or additive-proportional) to account for residual variability, and stepwise estimation of inter-individual variability (IIV) for each PK parameter.

Subsequently, saliva drug concentration-time data were added to the stable plasma model, by testing either a separate saliva compartment or a “scale” parameter assigned to the plasma compartment (Wicha et al., 2019). Separate error models were used to account for residual variability of the saliva drug concentrations.

Model selection was based on a significant decrease in objective function value (OFV, −2log likelihood) of at least −3.84 (p < 0.05, *χ*^2^ distribution) for each parameter change, for nested models. Akaike information criterion (AIC) was used for non-nested models. Evaluation of the model fit was performed using diagnostic plots (observed vs. predicted concentrations, residual error vs. predicted concentrations) and simulation-based diagnostics such as visual predictive checks (VPCs) and bootstrap to assess parameter uncertainty. Potential covariates were tested on the parameters, either by stepwise covariate modeling (scm) or manual inclusion in the model (Eq. 1 and Eq. 2), depending on the size of the dataset (Ribbing and Jonsson, 2004).

(Eq. 1) or, $$\text{P}=\theta_{\text{p}}\text{ x }(\text{COV}/\text{COV}\_\text{median})\;(\text{e.g., for weight})$$

(Eq. 2) $$\text{P}=\theta_{\text{p}}\text{ x }{\left(\text{COV}/\text{COV}\_\text{median}\right)}^{\theta \text{cov}}$$

where P is an individual value for a PK parameter, θ_p_ is the typical population value for a PK parameter, and θ_cov_ is the power exponent defining the covariate relationship, COV is a covariate type, and COV_median is the median value for the covariate.

## Results

### Systematic Literature Review

**Figure 1:**
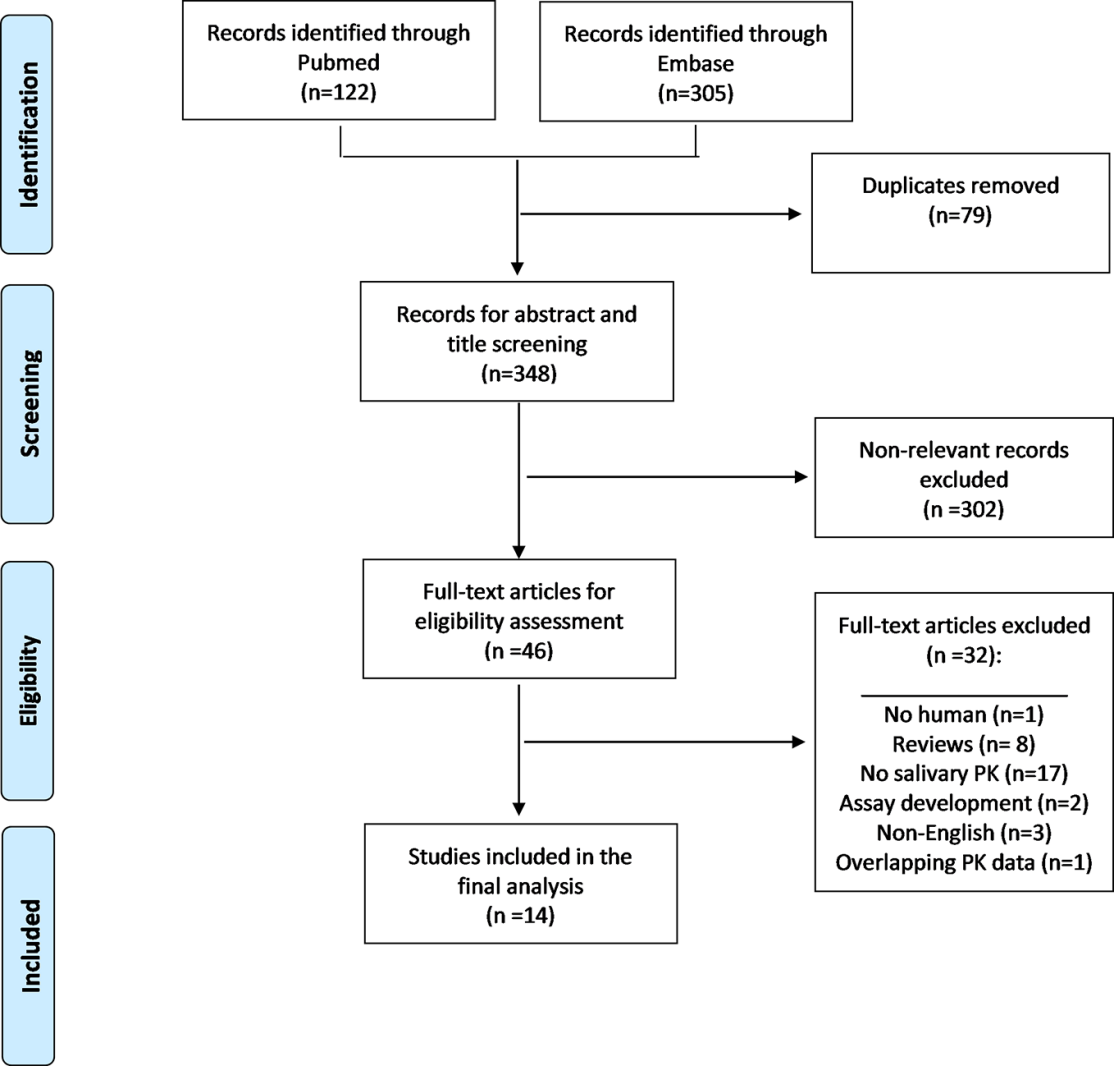
Flow diagram for the systematic review.

The search retrieved a total of 427 articles (n = 122 Pubmed, n = 305 Embase), resulting in 348 articles after removal of 79 duplicates (**Figure 1**). After excluding non-relevant references through title and abstract screening, 46 full text articles were assessed for eligibility. A total of 32 articles were excluded for reasons such as non-human data, reviews, non-English, no salivary drug concentrations or saliva-based HPLC assay development without saliva plasma concentration-time profiles. One study (Wildfeuer et al., 1996) was excluded due to overlapping PK data with another included study (Laufen et al., 1995). References of the eight excluded review articles were also screened, and no additional studies were identified. A total 14 studies were selected for inclusion in the systematic review. Of these, eight studies were conducted on fluconazole, one of which was also conducted in ketoconazole, four studies on voriconazole, two studies on itraconazole. No studies were identified for isavuconazole, posaconazole, flucytosine, amphotericin B, caspofungin, micafungin, or anidulafungin.

#### Fluconazole

From the eight studies, four studies were conducted in healthy volunteers (Oliary et al., 1993; Force and Nahata, 1995; Laufen et al., 1995; Koks et al., 1996), three studies were conducted in HIV (+/−AIDS) patients (Garcia-Hermoso et al., 1995; Koks et al., 1995; Koks et al., 2001), and one study was conducted in hospitalized children (van der Elst et al., 2014) (**Table 1**). Fluconazole dose in these studies ranged from 50–200 mg oral (po) daily, and median 9.4 mg/kg/day po or intravenous (iv) for children (van der Elst et al., 2014).

**Table 1:** Fluconazole studies included in the systematic review.

Author, year	Study population (N)	Study type	Dose, Duration	Sampling times	Saliva sampling + stimulation	Analytical method	Saliva C_max_ (mg/L), AUC_0-24_ (mg.h/L)	Plasma C_max_ (mg/L), AUC_0-24_ (mg.h/L)	S/P ratio, correlation	S/P ratio based on	Support saliva TDM (Y, I, N, N/A)^a^
Oliary et al. (1993)	HV, N = 5	Cohort study	50 mg po, single	0, 1, 1.5, 2, 4, 6, 10, 24 h	suction+ electrical	HPLC-UV	1.60 ± 0.24, 26.8 ± 7.0	1.15 ± 0.15, 20.7 ± 3.0	1.30 ± 0.29	AUC_0-24_	N/A
	Patients (salivary gland area irradiated), N = 5		50 mg po, single	0, 1, 1.5, 2, 4, 6, 10, 24 h	suction+ electrical	HPLC-UV	2.04 ± 0.39, 30.8 ± 5.9	1.40 ± 0.39, 22.9 ± 8.4	1.41 ± 0.30	AUC_0-24_	N/A
Force and Nahata (1995)	HV, N = 8	Randomized cross over	10 0mg po, single	0, 1, 2, 3, 6, 12, 24 h	tubes+ chewing paraffin	HPLC	2.56 ± 0.34 _	4.39 ± 3.33 _	0.55	C_max_	I
Garcia-Hermoso et al. (1995)	AIDS patients, N = 16	Prospective, observational	50–200 mg po daily, ≥7 days	0, 4 h	sterile tubes, disk	bio-assay (zone of inhibition)	7.73 ± 2.86^b^ _	6.74 ± 3.77^b^ _	1.26 ± 0.35^b^	C_max_	I (with MIC)
Laufen et al. (1995)	HV, N = 12	Cross-over formulation study	100 mg po, single (capsules)	0.1, 0.25 0.5, 1, 2, 3, 4, 6,8, 12, 24, 48, 72, 96 h	unstimulated	GC-ECD	3 ± 0.8, AUC_0-96_: 123.5 ± 25.5	2.5 ± 0.6, AUC_0-96_: 107.5 ± 21.1	1.19	C at multiple time-points (t = 4–24)	N/A
			100 mg po, single (suspension)	0.1, 0.25 0.5, 1, 2, 3, 4, 6, 8, 12, 24, 48, 72, 96 h	unstimulated	GC-ECD	551.1 ± 425.6^c^, AUC_0-96_: 227.7 ± 73.8	2.7 ± 0.7, AUC_0-96_: 105.2 ± 21.1	1.22	C at multiple time-points (t = 4–24)	N/A
Koks et al. (1995)	HIV patients with dry mouth, N = 1	Clinical applicability of assay	100 mg po, daily	0, 0.25, 0.5, 1, 1.5, 3, 5 h (at steady state)	Salivette^®^+ citric acid	HPLC-UV	9.5^d^ _	6.8 ^d^ _	T_0.5_ = 0.72, T_1.5_ = 1.45, T_3_ = 1.40^c^	C_0.5_, C_1.5,_ C_3_	N/A
Koks et al. (1996)	HV, N = 10	PK study	50 mg po bd on day 4 (capsules)	0, 0.25, 0.5, 1, 1.5, 2, 3, 4, 5, and 24 h	Salivette^®^+ citric acid	HPLC-UV	3.55 ± 0.40, 69.27 ± 12.89	_ 76.76 ± 13.83	T_0_ = 0.96 ± 0.33, T_3_ = 1.00 ± 0.24, T_5_ = 1.01 ± 0.27	C_0_, C_3,_ C_5_	N/A
			100 mg po on day 3 (suspension)	0, 0.25, 0.5, 1, 1.5, 2, 3, 4, 5, and 24 h	Salivette^®^+ citric acid	HPLC-UV	97.99 ± 6.12^e^, 89.13 ± 23.42	_ 76.91 ± 16.60	T_0_ = 1.16 ± 0.54, T_3_ = 1.04 ± 0.39, T_5_ = 1.09 ± 0.45	C_0_, C_3,_ C_5_	N/A
Koks et al. (2001)	HIV patients, N = 22	Prospective, observational	50 mg or 100 mg po daily, 7 days	3 h (median) (95% CI: 1.0–8.0) on day 7	Salivette^®^+ citric acid	HPLC-UV	_^f^	_^f^	1.3 (95% CI, 0.3–2.0) (r^2^ = 0.80, p < 0.001)	C at multiple time-points	I
van der Elst et al. (2014)	Hospitalized children, N = 19	Assay+ Clinical validation	9.4 mg/kg/ day (median) oral or IV, 31 days (median)	steady state C_min_ (trough)	Salivette^®^ or suction	LCMS/MS	C_min_: 3.5–27.5^g^	C_min_: 2.8–37.5	0.99 (95% CI, 0.88 to 1.10), (r = 0.960, p < 0.01)	C_min_	Y

^a^as concluded by the authors of the study; ^b^average values calculated from the data presented in the study; ^c,e^high C_max_ value due to the residual drug in the oral cavity from using suspension; ^d,g^estimated from the graph; ^f^median concentrations reported for multiple timepoints; C_max_ and AUC values are in mean ± SD, unless stated otherwise. Y, I, N, N/A; yes, intermediate (limited data), no, not applicable (authors do not mention saliva-based TDM); HV, healthy volunteers; po, oral dose; iv, intravenous; bd, twice daily; HPLC, high performance liquid chromatography; GC-ECD, gas chromatography-electron capture detector; HIV, human immunodeficiency virus; AIDS, acquired immunodeficiency syndrome; LCMS/MS, liquid chromatography-mass spectrometry.

Overall, fluconazole demonstrated a good penetration into saliva. S/P concentration ratios for fluconazole based on total drug were 1.21 on average (± 0.31 SD, range: 0.99–1.45), with exception of the study by Force and Nahata (1995), who reported a lower ratio of 0.55. In their study, no citric acid stimulation during saliva collection was used unlike in the three studies by Koks et al. (1995; 1996; 2001), although Laufen et al. report S/P ratio of 1.19 after unstimulated saliva collection (Laufen et al., 1995).

Fluconazole is a drug with a half-life of about 30 h, steady state is reached by day 7 with daily dosing (Brammer et al., 1990). In adults, S/P ratio after a single dose was 1.28 on average, across the studies (Oliary et al., 1993; Force and Nahata, 1995; Laufen et al., 1995), which is comparable to the average steady state ratio of 1.24 (Garcia-Hermoso et al., 1995; Koks et al., 1995; Koks et al., 1996; Koks et al., 2001), although sampling times were variable between the studies. Most S/P ratios for steady-state sampling were based on C_max_ or multiple time-points during the dosing interval. Lower S/P ratios were observed in children (0.99, 95% CI 0.88–1.10), although, this was based on trough concentrations, potentially resulting in values at the lower range (van der Elst et al., 2014).

Salivary drug exposure (AUC_0-96_) after fluconazole suspension was up to 80% greater (Laufen et al., 1995) compared to capsules, despite comparable plasma concentrations (Koks et al., 1996). This is likely due to the residual drug in oral cavity, over-estimating peak concentrations, rather than distribution from plasma to saliva (Laufen et al., 1995; Koks et al., 1996). S/P ratios became comparable after approximately 3–4 h post-dose in these studies (Laufen et al., 1995; Koks et al., 1996), with linear correlation between 4 to 24 h post-dose (Laufen et al., 1995).

In addition to excellent saliva penetration, three studies also reported significant correlation between the saliva and plasma fluconazole concentrations (Laufen et al., 1995; Koks et al., 2001; van der Elst et al., 2014), including consistent results in children (r = 0.96, p < 0.01) (van der Elst et al., 2014). Of these, van der Elst et al. strongly supported saliva-based fluconazole TDM (van der Elst et al., 2014), whereas Koks et al. recommended it as a semi-quantitative guide or for compliance monitoring, due to inadequate precision (prediction error of 55.8%) in their small dataset (Koks et al., 2001). TDM was not the subject of discussion for Laufen et al. (1995).

#### Voriconazole

From the four studies investigating saliva PK of voriconazole, two studies were conducted in healthy volunteers (Purkins et al., 2002; Purkins et al., 2003), one study in adult patients (Vanstraelen et al., 2015), and one study in adult and pediatric patients (Michael et al., 2010) (**Table 2**). Voriconazole dose in adults ranged from 3–5 mg/kg po (tablet or capsules) or iv twice daily with or without loading, and 7 mg/kg iv twice daily in children. Treatment period ranged from 4 to 14 days, and all four studies included collection of steady state samples at multiple time-points up to 12 h post-dose.

**Table 2:** Voriconazole studies included in the systematic review.

Author, year	Study population (N)	Study type	Dose, Duration	Sampling times	Saliva sampling + stimulation	Analytical method	Saliva C_max_ (mg/L), AUC_0-24_ (mg.h/L)	Plasma C_max_ (mg/L), AUC_0-24_ (mg.h/L)	S/P ratio, correlation	S/P ratio based on	Support saliva TDM (Y, I, N, N/A)
Purkins et al. (2002)	HV (male), N = 42	Dose-escalation cohort study	Loading + 3 mg/kg, 4 mg/kg or 5 mg/kg iv bd, then po bd, 14 days (capsules)	day 7 (iv), day 14 (po), frequently up to 12h	tube+ PTFE tape stimulation	HPLC-UV	range of medians for iv:^h^ 2.1–4.3, 8.6-25.0 po: 1.3–3.3, 6.0-22.0	range of medians for iv:^h^ 3.0–7.2, 14.0–43.1 po: 1.9–5.3, 9.8-37.5	0.66 (CV30%) (iv), 0.64 (CV29%) (po)	C at multiple time-points	Y
Purkins et al. (2003)	HV, N = 12	Single-blind, randomized cohort study (saliva data in Study A)	No loading, 3mg/kg iv daily (day1,12), bd (day 3–11)	day 1 and at steady state (day 12), up to 12h post-dose	tube+ PTFE tape stimulation	HPLC-UV	2.26^i^, AUC_0-12_: 10.911	3.621, AUC_0-12_: 16.535	0.62 (day1), 0.66 (day12)	AUC_0-12_	Y
Michael et al. (2010)	Children (N = 7), Adult (N = 9) patients	Prospective, observational PK study	Children: 7mg/kg iv bd, 10 days.	day 1-10, C_min_ (trough)	Salivette^®^+ citric acid	HPLC-fluorescence	Children: C_min_: 1.2	Children: C_min_: 2.8	Children: 0.34 (CV23%) (r = 0.98, p <0.001)	C_min_	Y
			Adults: Loading+4mg/kg iv bd, 10 days.				Adults: C_min:_ 0.6	Adults: C_min:_ 1.7	Adults: 0.4 (CV24%) (r = 0.95, p <0.001)		
Vanstraelen et al. (2015)	Patients, N = 10	Prospective, observational PK study	3.7 ± 0.4 mg/kg po (tablet) or iv bd, ≥ 4 days	0, 0.5, 1, 1.5, 2, 6 and 12 h	Salivette^®^	LCMS/MS	3.3 (2.7–4.2) (median, IQR), AUC_0-12_: 23.9 (15.8–32.1)	6.0 (4.0-9.3) (total drug), 2.9 (2.0-4.8) (unbound), AUC_0-12_: 47.0 (28.7–66.6) (total), 23.2 (14.2–34.2) (unbound)	0.51 ± 0.08 (r = 0.891, p < 0.001) (total drug)^j^ 0.49 ± 0.03 (r = 0.970, p < 0.001) (unbound)	C at multiple time-points	Y

^h^each dose cohort had median (range) reported in the study; for iv (3, 4, 5 mg/kg) and po (200, 300, 400 mg); ^i^values from day 12 (steady state); ^j^linear upto 10mg/L of total drug plasma concentration. PTFE, polytetrafluoroethylene.

S/P ratios for voriconazole were comparable between the studies with average value of 0.56 (± 0.18 SD, range: 0.51–0.66), although Michael et al., reported lower S/P ratio in children compared with adults (0.34 vs. 0.4, p = 0.014) (Michael et al., 2010). The ratios in the studies were based on AUC_0-12,_ C_min_ (trough) or concentrations at multiple time-points during dosing interval. The majority of studies reported significant correlation between saliva and plasma concentrations (r = 0.89 to 1.08, p < 0.0001 or < 0.001) in both adults (Purkins et al., 2002; Vanstraelen et al., 2015) and children (Michael et al., 2010). Vanstraelen et al. observed improved linear correlation for free voriconazole concentration (S/P ratio 0.49 ± 0.03, r = 0.970, p < 0.001), compared with total drug concentration. However, the correlation remained strong (S/P ratio 0.51 ± 0.08, r = 0.891, p < 0.001) up to 10 mg/L total voriconazole plasma concentration (Vanstraelen et al., 2015).

Only Purkins et al. investigated voriconazole exposure-toxicity associations (Purkins et al., 2002). However, the differences they observed in visual disturbances (29% vs. 21%), and liver enzyme elevations (two patients vs. none) in high-dose cohort compared to low-dose cohort did not reach a statistical significance, likely due to the small study size (n = 14) (Purkins et al., 2002).

Overall, saliva-based voriconazole TDM was supported by the authors of all four studies (Purkins et al., 2002; Purkins et al., 2003; Michael et al., 2010; Vanstraelen et al., 2015), and was the main subject for investigation by Michael et al. (2010) and Vanstraelen et al. (2015).

#### Itraconazole

Two studies investigated salivary PK of itraconazole (Reynes et al., 1997; Cross et al., 2000) (**Table 3**). S/P ratios were based on multiple concentrations during the dosing interval at steady state, after an oral dose of 100 mg twice a day. Itraconazole suspension resulted in highly variable S/P ratios in both studies, largely due to a topical effect. Cross et al. reported a median S/P ratio of 0.115 for itraconazole suspension, with a high variability (range: 0–3.71) (Cross et al., 2000). In the same study, itraconzole concentrations were undetectable in saliva, after the use of capsules, confirming a lack of itraconzole pentration into saliva (Cross et al., 2000). Reynes et al. also reported inconsistent S/P ratios due to high variability in itraconazole saliva concentrations (1.64 ± 2.05, mean ± SD) after the use of suspension (Reynes et al., 1997). No active metabolite, hydroxy-itraconazole, was detected in saliva (Reynes et al., 1997).

**Table 3:** Itraconazole and ketoconazole studies included in the systematic review.

Author, year	Study population (N)	Study type	Dose, Duration	Sampling times	Saliva sampling + stimulation	Analytical method	Saliva C_max_ (mg/L), AUC_0-24_ (mg.h/L)	Plasma C_max_ (mg/L), AUC_0-24_ (mg.h/L)	S/P ratio, correlation	S/P ratio based on	Support saliva TDM (Y, I, N, N/A)
**Itraconazole**											
Reynes et al. (1997)	HIV patients (+/− AIDS), N = 23	Prospective cohort PK study	Suspension, 100mg po bd, 14 days	0, 2, 4, 8 h on day 1 & day 14	syringe	Reverse-phase HPLC	without AIDS: 1.64 ± 2.05^k^, _ with AIDS: 4.07 ± 3.91 _ (no active metabolite detected in saliva)	without AIDS: 0.95 ± 0.38, AUC_0–10_: 7.78 ± 3.14 with AIDS: 0.70 ± 0.39, AUC_0–10_: 6.25 ± 3.86	Variable	C at multiple time-points (day 14)	N
Cross et al. (2000)	Patients, N = 40	Randomized, formulation comparison study	Suspension, 100mg po bd, 15 days	random, day 15 (median t = 4 h)	tubes	Reverse-phase HPLC	C random times, 0.12 (IQR 0–0.53)	C random times, 0.74 mg/L (IQR 0.46–1.18)	0.115 (0-3.71)^l^	C at multiple time-points, day 15	N
			Capsules, 100mg po bd, 15 days	random, day 15 (median t = 3 h)	tubes	Reverse-phase HPLC	not detected^m^	C random times, 0.61 (IQR 0.37–0.93)	0	C at multiple time-points, day 15	N
**Ketoconazole**											
Force and Nahata (1995)	HV, N = 8	Randomized cross over (with fluconazole) study	400mg po, single (tablet)	0, 1, 2, 3, 6, 12, and 24 h	tubes	HPLC	0.119 ± 0.050 _	7.64 ± 3.87 _	0.011	C_max_	N

^k^note high variability (high SD), explains no statistically significant differences between patients with vs. without AIDS; ^l^calculated from data presented in the study;

^m^two patients had aberrantly high saliva concentrations, which were considered due to sampling or analytical error; C, concentration.

#### Ketoconazole

Salivary PK of ketoconazole was reported in one study, comparing fluconazole and ketoconazole penetration into saliva and clinical efficacy in the treatment of oropharyngeal-esophageal candidiasis (Force and Nahata, 1995) (**Table 3**). This study was conducted in eight healthy volunteers after a single oral dose of 400 mg (tablet), with saliva samples collected up to 24 h post-dose. S/P ratio, based on C_max,_ was 0.01 at 2 h post-dose, and ketoconazole saliva concentration was undetectable at 24 h post-dose.

### Population PK Analysis

#### Data Retrieval From Authors' Studies

Although both fluconazole and voriconazole qualified for further analysis and saliva population PK model development, voriconazole had the stronger evidence for saliva-based TDM. Furthermore, after contacting the authors for access to individual patient data, no data could be retrieved for fluconazole, as most papers were published over two decades ago.

For voriconazole, data could be retrieved from one study reporting correlation of saliva and plasma voriconazole concentrations in patients admitted to adult oncology/haematology and respiratory wards or paediatric ward for treatment of invasive aspergillosis (Vanstraelen et al., 2015). A total of 137 saliva (n = 68) and plasma (n = 69) voriconazole concentrations were available from 11 patients (10 adults, 1 child) for PK analysis (Vanstraelen et al., 2015). The paediatric patient (age 9 years old), excluded in the original study was included in our PK analysis after excluding one outlier saliva concentration (Vanstraelen et al., 2015). Seven patients were male, and four patients were female. The median age in adults was 55 years (range 30–66) with a mean body weight of 65.9 ± 20.1 kg. The patients were on either oncology/haematology (eight patients) or respiratory (three patients) ward. Detailed patient characteristics are also outlined in the authors' publication (Vanstraelen et al., 2015). In the study, the patients were treated with voriconazole (iv or oral, every 12 h) for at least 4 days, and saliva and plasma samples were collected at pre-dose, 0.5, 1, 1.5, 2, 6, and 12 h post-dose (Vanstraelen et al., 2015). Saliva and plasma voriconazole concentrations were measured with liquid chromatography–tandem mass spectrometry, with intra- and inter-day precision of 1.2–3.5% and accuracy of 5.5–5.9% for saliva, and precision of 2.6–7.6% and accuracy of −0.5–1.21% for plasma (Vanstraelen et al., 2015).

#### Salivary Pharmacokinetics of Voriconazole

Plasma pharmacokinetics of voriconazole was better described by a one-compartment model with the first-order absorption and first-order elimination, compared to a two-compartment model, as determined by model stability in parameter estimates, RSE (residual standard error), and diagnostic plots. A proportional error model (ΔOFV, −54.0) better accounted for residual variability in plasma voriconazole concentrations compared to an additive error model. Use of a combined (proportional + additive) error model resulted in no additional improvement in objective function value (ΔOFV, −0).

**Figure 2:**
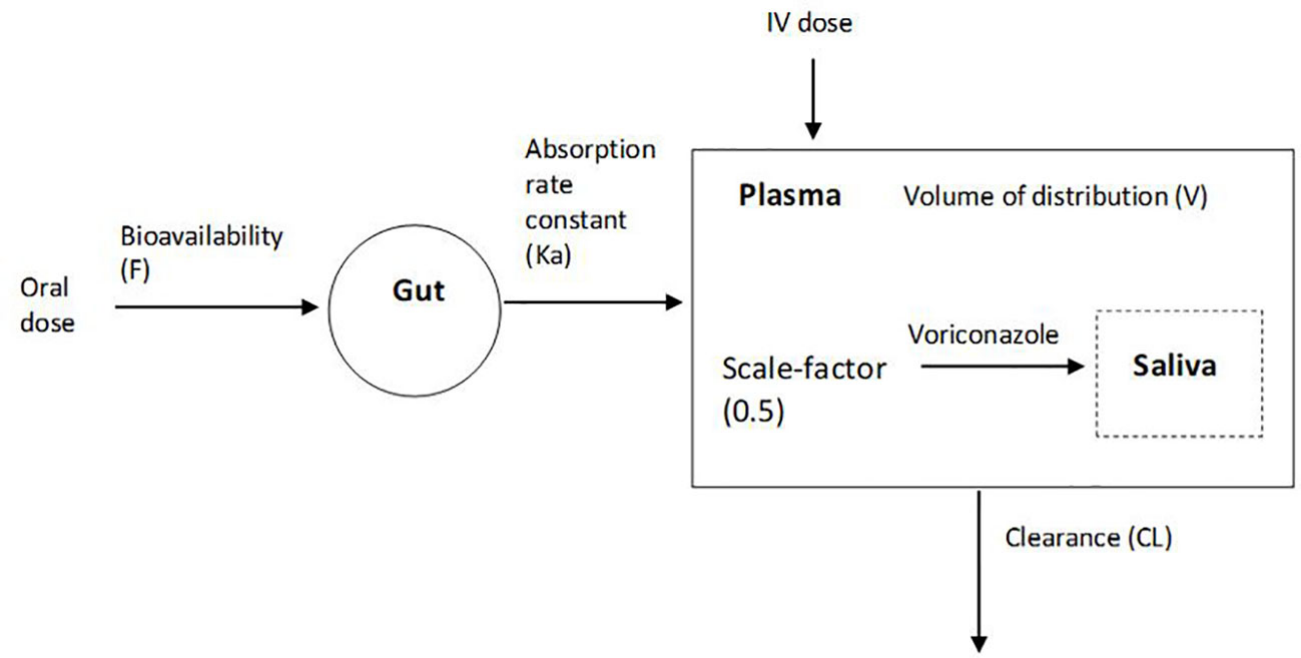
Schematic representation of the developed 1-compartment voriconazole PK model, with a scale-factor for plasma to saliva voriconazole distribution.

Saliva voriconazole concentrations were added to the stable plasma model. Use of a scale-factor to the plasma compartment (**Figure 2**) significantly improved the model fit (ΔOFV, −102.7) compared to a separate saliva compartment. Residual variability for saliva voriconazole concentrations was better accounted for using a proportional error model (ΔOFV, −24.9) compared to an additive error model. Use of a combined (proportional + additive) error model resulted in no additional improvement in OFV (ΔOFV, +0.001), therefore a proportional error model was selected for the saliva part of the model as well. Absorption rate constant (Ka) was fixed to the final estimate (0.858) to increase model stability due to high RSE (129%) when estimated. The final one-compartment PK model describing voriconazole in saliva and plasma simultaneously was parameterized by clearance (CL) of 4.56 L/h with IIV of 36.9%, volume of distribution (V) of 60.7 L, and bioavailability (F) of 0.849 (**Table 4**). IIV for V could not be estimated due to model instability resulting in high shrinkage (99%). Due to the small dataset and for the purpose of focusing on characterization of saliva drug distribution, stepwise covariate modeling was not performed, as this can lead to selection bias, and falsely positive covariate identification (Ribbing and Jonsson, 2004). Known covariates for voriconazole were individually tested on CL. Inclusion of covariates on CL, including weight (WT/70 kg) (ΔOFV, +6.15), weight with allometric scaling (WT^0.75^/70kg) (ΔOFV, +11.39), AST (ΔOFV, +14.49), ALT (ΔOFV, +12.48), ALP (ΔOFV, +18.99), and bilirubin (ΔOFV, +10.68), did not result in significant improvement in OFV.

**Table 4:** Population PK parameters (θ), Inter-individual variability (ω^2^), Residual variability (σ^2^) from the final model simultaneously describing saliva and plasma voriconazole pharmacokinetics.

PK parameter	Final model estimate	RSE (%)	Bootstrap estimate (n = 1,000) (median [95% CI])	Bias (%)
Clearance (CL) = θ_1_ (L/h)				
θ_1_	4.56	16%	4.39 [3.23–5.98]	3.7%
ω^2^	0.136 (36.9% CV)	42%	0.115 [0.027–0.230]	15.4%
Central volume of distribution(V) = θ_2_ (L)				
θ_2_	60.7	12%	57.9 [41.4–72.3]	4.6%
ω^2^	–	–		
Absorption rate constant (Ka) = θ_3_ (t^−1^)				
θ_3_	0.858 (fixed to model estimate)	–	0.858 (fixed)	–
ω^2^	–	–	–	
Bioavailability (F) = θ_4_				
θ_4_	0.849	14%	0.819 [0.577–0.983]	3.5%
ω^2^	–	–	–	
Scale-factor (Scale) = θ_5_				
θ_5_	0.501	4%	0.499 [0.458–0.541]	0.4%
ω^2^	–	–	–	
Residual variability model				
σ^2^_Proportional,_ _Plasma_	0.057	25%	0.054 [0.032–0.083]	5.3%
σ^2^_Proportional,_ _Saliva_	0.078	26%	0.074 [0.041–0.112]	5.1%

RSE, relative standard error; CI, confidence interval; CV, coefficient of variation.

**Figure 3:**
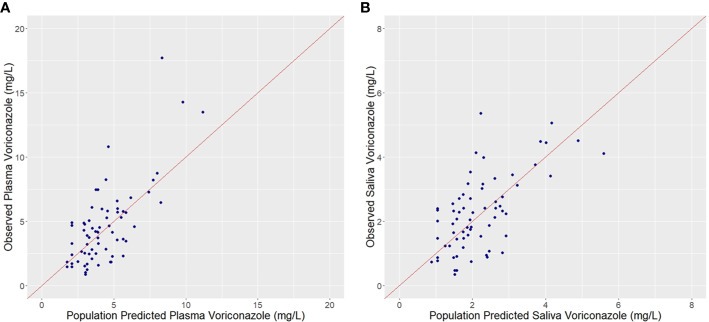
Observed vs. Population predicted plasma **(A)** and saliva **(B)** voriconazole concentrations.

**Figure 4:**
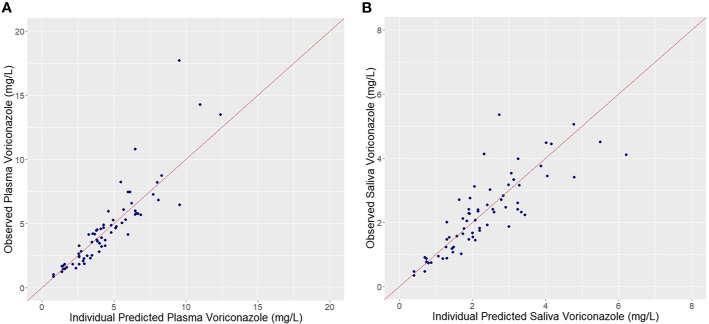
Observed vs. Individual predicted plasma **(A)** and saliva **(B)** voriconazole concentrations.

**Figure 5:**
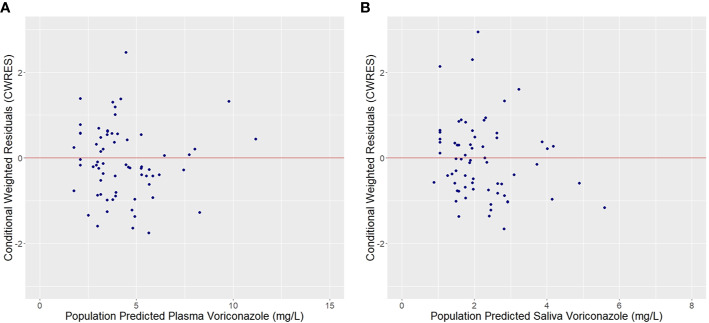
Conditional weighted residuals (CWRES) vs. Population predicted plasma **(A)** and saliva **(B)** voriconazole concentrations.

**Figure 6:**
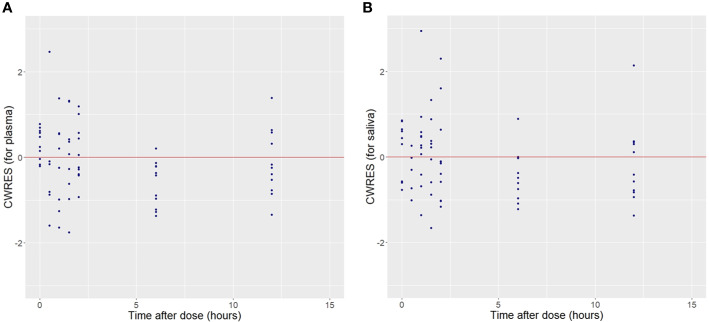
Conditional weighted residuals (CWRES) for plasma **(A)** and saliva **(B)** voriconazole observations vs. Time after dose.

**Figure 7:**
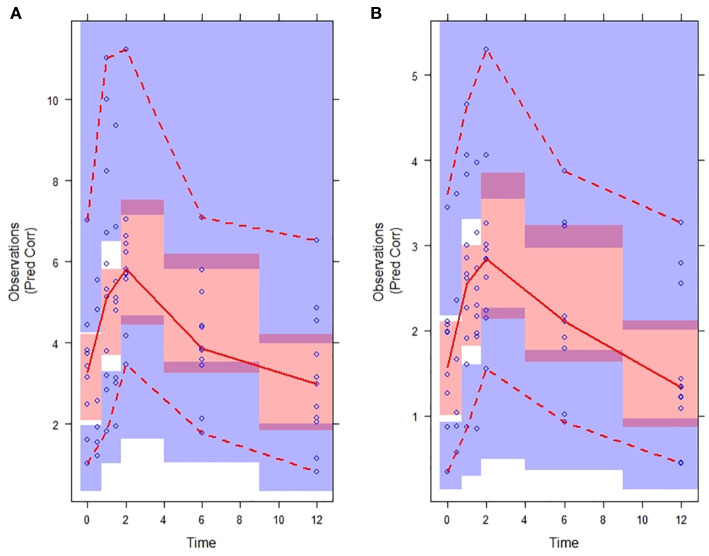
Predicted-correlated visual predictive check (VPC) of the final voriconazole PK model stratified on plasma **(A)** and saliva **(B)**. Time in hours. Observations: voriconazole concentrations (mg/L). Observed voriconazole concentrations (blue circles) with median (red solid line) and 5^th^ and 95^th^ percentiles (red dotted lines). Simulated (n = 1,000) voriconazole concentrations with 95% confidence interval of the median (red shade), 5^th^ and 95^th^ percentiles (purple shade).

Plots of observed vs. population predicted or individual predicted voriconazole concentrations (**Figures 3** and **4**) showed a good fit of the model to the data. Residual plot of conditional weighted residual (CWRES) vs. population predicted voriconazole concentrations showed relatively even distribution around y = 0 line, indicating no model misspecification (**Figure 5**). CWRES vs. time after dose plot also confirmed no time-dependent residual errors (**Figure 6**). Bootstrap analysis (simulation, n = 1,000) showed comparable median and confidence intervals for the simulated predictions and confirmed minimal model uncertainty (**Table 4**). One hundred forty-nine bootstrap runs with estimates near a boundary were skipped. VPC (predicted-corrected simulations, n = 1,000) also confirmed good predictive performance of the final model, as the predicted central tendency and intervals were in line with the observed voriconazole concentrations (**Figure 7**).

## Discussion

In this study, the systematic review evaluated evidence for saliva-based TDM of antifungal drugs. Voriconazole and fluconazole demonstrated susbstantial saliva penetration. Strong correlation between saliva and plasma drug concentrations were observed for both drugs based on total drug exposure or concentrations at multiple timepoints after dose. The authors of the included studies strongly supported saliva-based TDM for voriconazole, whereas fluconazole studies lacked investigation of saliva-based TDM as the subject matter.

We developed a saliva population PK model for voriconazole based on the S/P PK data, kindly provided by Vanstraelen et al. (2015). The kinetics of the plasma to saliva voriconazole distribution was identical to the kinetics of voriconazole in plasma; however, the extent of distribution was lower, described by the model-estimated scale factor of 0.501.

The systematic review identified limited studies which investigated the salivary PK of the four antifungal drugs, including fluconazole, voriconazole, itraconazole, and ketoconazole. No saliva data was available for other antifungal drugs, likely due to a lack of rationale for concentration-guided dosing (Ashbee et al., 2014; Patterson et al., 2016; Andes et al., 2018; Maertens et al., 2018; John et al., 2019; Warris et al., 2019).

Excellent saliva penetration of fluconazole (S/P ratio 1.21 on average) observed in our review is supported by chemical and molecular properties of fluconazole such as low protein binding (12%), unionized state of the drug in physiological conditions (pH 6–7) and hydrophobicity, which allow crossing of lipophilic salivary gland membranes. Penetration of fluconazole into other bodily tissues and fluids has also been reported in early studies (Brammer et al., 1990). Interestingly, fluconazole S/P ratios reported in the majority of the included studies were greater than 1, indicating greater saliva concentrations compared to actual plasma concentrations. The underlying mechanisms suggested were fluconazole binding to saliva constituents or due to drug trapped in saliva as a result of ionization resulting from low pH when citric acid is used for saliva stimulation (Koks et al., 2001). This could also explain why Force and Nahata (1995) observed lower average S/P ratio in their patients as no citric acid stimulation was used unlike in the three studies by Koks et al. (1995; 1996; 2001).

Similarly, voriconazole demonstrated substantial saliva penetration (S/P ratio 0.56 on average) and strong correlation between saliva and plasma concentrations in the studies. Voriconazole demonstrates non-linear, saturable PK, with dose increase resulting in supra-proportional increase in exposure (Purkins et al., 2002; Purkins et al., 2003; Dolton and McLachlan, 2014). Voriconazole S/P ratio was consistent for low, medium, and high dose cohorts (Purkins et al., 2002). Unlike fluconazole, voriconazole salivary distribution is not affected by pH and citric acid stimulation, as it is weakly basic with pKa 1.76 (Michael et al., 2010). Voriconazole saliva concentrations were generally lower than plasma concentrations, resulting in S/P ratios of 0.56 on average for adults across the studies. Salivary distribution of voriconazole also showed a strong correlation with plasma distribution in children (Michael et al., 2010). Overall, in both children and adults, the S/P ratios generally well reflected unbound fraction of the drug in plasma (42%) (Michael et al., 2010), supporting Vanstraelen et al. who reported stronger S/P correlation for unbound voriconazole compared to the total drug (Vanstraelen et al., 2015).

In contrast to fluconazole and voriconazole, ketoconazole, and itraconazole had poor evidence supporting the salivary penetration. High protein binding of both ketoconazole (99%) and itraconazole (99.6%), and lipophilic nature of itraconazole (Felton et al., 2014) provide explanations for poor penetration into saliva. Indeed, the observed S/P ratio of 0.011 for ketoconazole correspond to the 1% unbound fraction of the drug, demonstrating low saliva penetration even at C_max_ (Force and Nahata, 1995).

Similarly, for itraconazole, only free fraction of the drug was detected in saliva, without the active metabolite, hydroxy-itraconazole (Reynes et al., 1997). Acid-dependent nature of itraconazole absorption (van der Elst et al., 2015) may explain low and variable bioavailability and saliva drug concentrations in the studies (Reynes et al., 1997; Cross et al., 2000). High inter-patient variability was observed for itraconazole salivary and plasma distribution in HIV patients (Reynes et al., 1997). Although Reynes et al. do not report on significant differences in itraconazole PK between HIV patients with or without AIDS, previous studies have reported reduced bioavailability and altered itraconazole PK in HIV patients with AIDS potentially due to gastric secretory failure (Smith et al., 1992).

Structural and chemical similarities (e.g., lipophilicity) of posaconazole to itraconazole, and high protein binding of posaconazole (>98%) and echinocandins (84–99.85%) (Hajdu et al., 1997; Paderu et al., 2007; Felton et al., 2014; Wasmann et al., 2018) may allow speculation for poor saliva penetration of these drugs.

Detection of misleadingly high drug concentrations after the use of suspension due to the residual drug in the oral cavity, suggest the importance of rinsing mouth before saliva sampling, and could further be studied as part of a validation of saliva-based TDM for relevant drugs in the future.

Evidence supporting routine fluconazole TDM is weak, although TDM could be beneficial in selected cases such as during renal dialysis, high-dose therapy for CNS infections (Ashbee et al., 2014; John et al., 2019). Fluconazole has excellent bioavailability and given the linear pharmacokinetics, its exposure is predictable from clinically relevant doses except in patients at risk of altered PK such as critically ill patients on haemofiltration (John et al., 2019). Also during treatment of less susceptible strains with high minimum inhibitory concentration (MIC), optimal exposure/susceptibility target (e.g., AUC/MIC) may not be attained, and fluconazole TDM may provide a benefit (Ashbee et al., 2014).

In this study, the literature review provided a strong rationale for exploring a saliva model for voriconazole. Dosing decisions based on optimal PK/PD target attainment is imperative for voriconazole to achieve treatment success. Saliva sampling can be a potential alternative to invasive plasma sampling, especially benefiting pediatric patients, and point-of-care saliva testing can allow short turnaround times and prompt dose adjustments, which are crucial for critically ill patients.

No studies investigated saliva modeling of voriconazole. The scale-factor identified in our population PK model, to describe voriconazole distribution from plasma to saliva, correlated well with the clinically reported S/P ratios (0.56) in the studies included in our systematic review. Inclusion of weight or liver function tests as covariates did not further improve the model. Commonly identified covariates from literature include weight, cytochrome P450 *2C19* genotype and liver function (e.g., ALT and AST) (Shi et al., 2019). Due to the high risk of selection bias, it is recommended to avoid stepwise covariate modeling for inclusion of statistically significant covariates in small data sets (<50–100 subjects), especially if the aim of the analysis is predictive modeling (Ribbing and Jonsson, 2004). In our study, the aim was to understand the salivary pharmacokinetics of voriconazole, and to develop a simple and predictive model, which represents a first step toward saliva-based precision dosing of voriconazole, together with clinical validation and external model evaluation and clinical validation. In addition, IIV was estimated for only CL parameter in our study, due to high model instability and uncertainty on parameter estimates, potentially due to the small dataset. Therefore, inclusion of covariates would not have been highly informative.

Previous plasma population models for voriconazole vary between the studies, in terms of PK compartments (one or two compartments), identified covariates, and PK parameter estimates (Shi et al., 2019). Amongst these studies, parameters from our study were comparable to those from Pascual et al. (Pascual et al., 2012), who fitted a one-compartment model with the first-order absorption and first-order elimination to 505 plasma voriconazole concentrations from 55 adult patients. The patient demographics in this study (Pascual et al., 2012) were also similar to our adult patients (Vanstraelen et al., 2015). A number of other studies conducted in adults (Pascual et al., 2012; Wang et al., 2014; Li et al., 2017; Lin et al., 2018) also fitted plasma voriconazole data to one-compartment model with the first-order absorption and first-order elimination, used in our model. Many of these studies identified *CYP2C19* genotype as a covariate on CL or V (Wang et al., 2014; Li et al., 2017; Lin et al., 2018). Availability of *CYP2C19* genotype in our study may have been useful in accounting for CL variability. Some studies also fitted voriconazole data to a one-compartment model, but with absorption lag time (Han et al., 2011), Michaelis-Menton elimination (Mangal et al., 2018) or first-order elimination (Nomura et al., 2008), or to a two-compartment model (Dolton et al., 2014). Greater IIV of voriconazole pharmacokinetics was observed in children compared to adults, and a higher weight-based dosing is recommended in adolescents to achieve comparable exposure to adults (Friberg et al., 2012). This could explain why majority of the studies conducted in paediatrics fitted more complex two-compartment models (Walsh et al., 2004; Karlsson et al., 2009; Friberg et al., 2012). Furthermore, such differences between the previous models for voriconazole plasma PK reflect variable voriconazole PK depending on patient population and characteristics.

In our study, concentration data from one paediatric patient was included for the purpose of maximizing the available dataset. The saliva model could be expanded in order to characterize salivary pharmacokinetics voriconazole in paediatric patients.

Limitations of our study include the small dataset, which can be expanded with future studies involving pilot prospective studies for voriconazole saliva-based TDM, and potentially expanding the modeling to paediatric population, who will benefit from noninvasive saliva sampling. Further limitations include availability of only steady state data, a single dose occasions not allowing estimation of inter-occasion variability, which is important to judge the feasibility of the model-informed precision dosing. Also, richer data would have allowed estimation of inter-patient variability or covariates for the salivary drug penetration, modeled by a scale-factor in our study.

Saliva population PK models, in addition to larger clinical validation of saliva assay or development of point-of-care saliva devices, will enable clinical implementation of saliva-based precision dosing.

Furthermore, saliva PK studies for newer antifungal drugs, and continued investigation of exposure-response relationships in relation to the pathogen's susceptibility and relevant PD markers (e.g., galactomannan monitoring) (Negri et al., 2018) may allow the use of saliva TDM for other antifungal drugs in the future.

## Conclusion

The systematic review showed that fluconazole and voriconazole had a good saliva drug penetration and a strong S/P correlation of drug concentrations. Voriconazole had a strong evidence to support saliva-based TDM. The developed population PK model was able to predict the salivary distribution of voriconazole using a scale factor assigned to the central plasma compartment. The saliva models of antifungal drugs have the potential to provide a framework for saliva-based TDM for both hospitalized patients as well as in the community setting to support model-informed precision dosing to optimize treatment of invasive fungal infections.

## Data Availability Statement

The datasets generated for this study are available on request to the corresponding author.

## Ethics Statement

The studies involving human participants were reviewed and approved by University Hospitals Leuven and the University Hospitals Brussels. Written informed consent to participate in this study was provided by the participants' legal guardian/next of kin.

## Author Contributions

HK conducted and completed the systematic review, data retrieval, PK modeling, and manuscript writing. J-WA contributed to the conception of ideas and overall supervision. SW contributed to PK model building and model optimization. A-GM provided scientific advices and support. IS and ED provided the voriconazole data. AM contributed by being an independent second assessor for the systematic search, study selection and data extraction. All authors contributed to the article and approved the submitted version.

## Funding

HK is supported by postdoctoral funding from the Sydney Pharmacy School, The University of Sydney. IS is supported by the Clinical Research Fund of the University Hospitals Leuven, Belgium. ED is a postdoctoral research fellow of the Research Foundation – Flanders (FWO), Belgium (grant number 12X9420N) and received consultancy fees from argenx and Janssen (all honoraria/fees paid to the department).

## Conflict of Interest

The authors declare that the research was conducted in the absence of any commercial or financial relationships that could be construed as a potential conflict of interest.
